# Capture of visual attention interferes with multisensory speech processing

**DOI:** 10.3389/fnint.2012.00067

**Published:** 2012-09-06

**Authors:** Hanna Krause, Till R. Schneider, Andreas K. Engel, Daniel Senkowski

**Affiliations:** ^1^Department of Neurophysiology and Pathophysiology, University Medical Center Hamburg-EppendorfHamburg, Germany; ^2^Department of Psychiatry and Psychotherapy Charité, University Medicine Berlin, St. Hedwig HospitalBerlin, Germany

**Keywords:** crossmodal, EEG, bimodal, SSVEP, oscillatory

## Abstract

Attending to a conversation in a crowded scene requires selection of relevant information, while ignoring other distracting sensory input, such as speech signals from surrounding people. The neural mechanisms of how distracting stimuli influence the processing of attended speech are not well understood. In this high-density electroencephalography (EEG) study, we investigated how different types of speech and non-speech stimuli influence the processing of attended audiovisual speech. Participants were presented with three horizontally aligned speakers who produced syllables. The faces of the three speakers flickered at specific frequencies (19 Hz for flanking speakers and 25 Hz for the center speaker), which induced steady-state visual evoked potentials (SSVEP) in the EEG that served as a measure of visual attention. The participants' task was to detect an occasional audiovisual target syllable produced by the center speaker, while ignoring distracting signals originating from the two flanking speakers. In all experimental conditions the center speaker produced a bimodal audiovisual syllable. In three distraction conditions, which were contrasted with a no-distraction control condition, the flanking speakers either produced audiovisual speech, moved their lips, and produced acoustic noise, or moved their lips without producing an auditory signal. We observed behavioral interference in the reaction times (RTs) in particular when the flanking speakers produced naturalistic audiovisual speech. These effects were paralleled by enhanced 19 Hz SSVEP, indicative of a stimulus-driven capture of attention toward the interfering speakers. Our study provides evidence that non-relevant audiovisual speech signals serve as highly salient distractors, which capture attention in a stimulus-driven fashion.

## Introduction

In everyday life, speech signals from a person that we are listening to are often accompanied by distracting other sensory input, such as auditory and visual stimuli from surrounding people. These distracting stimuli can capture attention and interfere with the recognition of speech. How exactly distracting auditory and visual speech stimuli affect the recognition and processing of attended speech is, to date, not well understood.

Speech recognition, in particular in noisy conditions, is considerably improved when matching visual inputs, i.e., lip movements, are presented (Sumby and Pollack, 1954; Ross et al., 2007a,b). Moreover, a recent functional magnetic resonance imaging study showed that attending to lip movements that match a stream of auditory sentences leads to an enhanced target detection rate and to stronger activity in a speech-related multisensory network compared to attending to non-matching lip movements (Fairhall and Macaluso, 2009). This suggests an important role of top-down attention for multisensory processing of speech (Koelewijn et al., 2010; Talsma et al., 2010).

This notion is consistent with an electroencephalographic (EEG) study, in which we examined the influence of task relevant and task irrelevant visual speech stimuli on audiovisual speech processing in a multiple speaker scenario (Senkowski et al., 2008). In this study, participants were instructed to detect an occasional audiovisual target syllable by a speaker (i.e., a speaking face) who was presented centrally and surrounded by two flanking speakers. The study comprised of *no interference* trials, in which a syllable was produced by the relevant central speaker only, and *interference trials*, in which different audiovisual syllables were produced by three speakers simultaneously. Using steady-state visual evoked potentials (SSVEP) as a real-time index of deployment of visual attention, we observed that visual attention toward the task irrelevant flanking speakers interferes with the recognition of task relevant audiovisual signals. The main open question raised by this study is whether the interference effect is specific for the processing of naturalistic audiovisual speech or whether similar effects would occur when the flanking speakers produce other distracting stimuli, like moving their lips without a sound or when they produce noise instead of syllables.

Using an extended setup of our previous study (Senkowski et al., 2008), we addressed this question by examining behavioral data and SSVEPs in three *interference* conditions and one control condition. In the interference conditions, the flanking speakers produced either naturalistic audiovisual syllables, lip movements alone, or lip movements in combination with acoustic noise. In line with our previous study (Senkowski et al., 2008), we expected distraction effects in behavioral data that are paralleled by enhanced SSVEPs to flanking speakers when these speakers produced naturalistic audiovisual speech. Given the salience of naturalistic audiovisual speech, we predicted that the interference effects of lip movements alone and lip movements accompanied by auditory noise would be much weaker or even vanished.

## Materials and methods

### Participants

Twenty volunteers, who reported no history of neurologic or psychiatric illness, participated in the study. Four participants were excluded from the analysis on the basis of extensive eye movements. Additional three participants were excluded because their hit rate (HR) was lower than 50% in the “Speech Interference” condition (see below). The remaining 13 participants (all right handed, mean age 22.92 years, range 21–29 years, 6 females) had normal hearing, as assessed by a hearing test in which 30 dB HL sinusoidal tones of varying frequencies had to be detected. Participants had normal or corrected-to-normal vision, as ensured by the Landolt test of visual acuity (visus ≥ 0.9). The Institutional Review Board of the Medical Association of Hamburg approved the experimental procedures, and each subject provided written informed consent and was paid for participation.

### Procedure and stimuli

**Figure 1:**
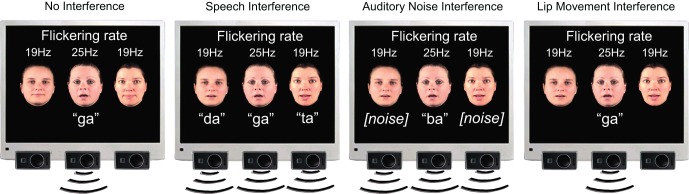
**Stimulus setup.** Stimuli consist of three horizontally aligned speakers on a black background. In all experimental conditions, the center face is visually presented in an on-off fashion so that a 25 Hz flicker was elicited. The center speaker produces natural auditory and visible syllables (‘ta’, ‘da’, ‘ga’, ‘ba’), whereas the two flanking speakers are always presented with a flicker frequency of 19 Hz. The subject‘s task is to detect the syllable ‘ba’ by the center speaker. In the No Interference condition, the flanking speakers produce neither visual lip movements nor speech sounds, whereas they produce natural speech syllables (‘ta’, ‘da’, and ‘ga’) simultaneous with the syllables of the center speaker in the Speech Interference condition. The Auditory Noise Interference condition consists of phase-scrambled versions of the original syllables ‘ta’, ‘da’, and ‘ga’ produced by the flanking speakers. In the Lip Movement Interference condition the flanking speakers produce lip movements of the original syllables without any accompanying auditory signal.

A continuous stream of four stimulation conditions was presented (Figure 1). Two of the conditions were identical to those used in our previous study (Senkowski et al., 2008). This previous study comprised of a “No Interference” control condition, in which only the center speaker produced a syllable, and a “Speech Interference” condition, in which all three speakers produced syllables (a short clip of this experiment is provided at: http://www.sciencedirect.com/science/article/pii/S1053811908007933). In the present study, two conditions were added to examine in further detail how visual attention toward flanking speakers interferes with audiovisual speech processing. In one of these conditions the flanking speakers produced acoustic non-speech noise instead of syllables. Non-speech noise samples were directly derived from the original syllables by phase-scrambling the auditory syllables, thereby maintaining basic properties like stimulus power. We will refer to this condition as “Auditory Noise Interference.” In the other condition the flanking speakers moved their lips without producing an acoustic syllable. We will refer to this condition as “Lip Movement Interference” condition. Thus, the study comprised of four conditions: “No Interference,” “Speech Interference,” “Auditory Noise Interference,” and “Lip Movement Interference.” The center speaker produced one of the syllables /ta/, /da/, /ga/, or /ba/ in all conditions, whereas the flanking speakers could produce the syllables /ta/, /da/, or /ga/ in the “Speech Interference” condition. The four conditions and the different syllables were presented in random order. Participants were instructed to focus their attention to the center speaker and to ignore the signals from the flanking speakers. Furthermore, they had to indicate the occasional appearance of the target syllable /ba/ by the center speaker with a button press of their right index finger. The target syllable occurred in 20% of all trials. The three speakers never produced the same syllable in a trial and syllable combinations that could evoke the McGurk illusion (McGurk and MacDonald, 1976), like the combination /ba/ and /ga/ were excluded.

On average 76 targets and 300 non-target stimuli were presented for each condition. One trial consisted of 120 visual frames of 6.67 ms each, resulting in a trial duration of 792 ms. Two fixed cycles of 24 frames were added per trial. Moreover, a variable number of 1–5 cycles (average: 3 cycles) was added, resulting in a total average trial duration of 1592 ms. During the inter-trial interval the faces of the three speakers were presented on the screen without producing any lip movements or speech sounds, but the 19 Hz flicker of the flanking speakers and the 25 Hz flicker of the center speaker continued. An additional number of 645 (about 30% of all trials) “omitted trial” periods (Busse and Woldorff, 2003) were randomly inserted into the continuous stream of stimuli, further reducing the predictability of the experimental stimuli. During omitted trial periods, the faces of the three speakers were presented for a time interval that was identical to the interval of regular experimental events (i.e., 792 ms) but without any lip movements or speech sounds. Each participant underwent 18 experimental blocks with 120 trials each.

Recordings of syllables from the three speakers were obtained at frame rates of 30/s. Each syllable consisted of 20 frames of 33 ms duration, which results in a total duration of 660 ms for each syllable. The visual angle of the speakers subtended 7° between adjacent speakers (from mouth to mouth) and the width of the speakers' faces subtended an angle of 4.8° each. The characters of the flanking speakers switched their location (i.e., left or right of the center speaker) after every block, while the center speaker character remained the same throughout the experiment. The monitor was set to a refresh rate of 150 Hz, i.e., the refresh rate duration for one frame was 6.67 ms. To induce SSVEPs, the continuous stream of pictures was dissected in an on–off fashion, i.e., pictures of the continuous stream (“on”) were presented alternately with blank screens (“off”). Pictures of the continuous stream and blank frames alternated every 20 ms. Thus, the flicker frequency (i.e., on–off cycle) was 25 Hz for the center speaker. For the two flanking speakers, the on–off periods alternated every 26.6 ms simultaneously for both speakers, corresponding to a flicker frequency of about 19 Hz. In the EEG the time-frequency (TF) transformed activity of a sustained visual on–off flicker is reflected in event-related activity that corresponds to the presented flicker frequency (Herrmann, 2001).

Both the 19 Hz flicker and the 25 Hz flicker were presented continuously and all trials started with an “on” period. The average stimulus duration of the acoustic syllables was 295 ms and the onset of these syllables followed the onset of visual lip movements on average by 230 ms. To eliminate overlapping event-related responses to the sounds, a relative stimulus onset jitter of 110 ms (more than two times the duration of a 19 Hz and a 25 Hz cycle) was used by adding or subtracting a random time interval between ±55 ms to the real acoustic sound onset in each trial (Woldorff, 1993; Senkowski et al., 2007). This jitter prevented overlapping event-related 19 and 25 Hz responses to the acoustic inputs. A spline curve FFT filter between 400 and 4000 Hz was applied to all syllables to align the voice characteristics between the three speakers.

### Data acquisition

The EEG was recorded from 124 scalp sites using an active electrode system (EASYCAP, Herrsching, Germany). In addition, the electrooculogram was recorded by two electrodes. One of these electrodes was placed below the eye and the other one was placed at the lateral bridge of the nose. The nose tip was used as reference during recording and data were off-line re-referenced to average reference. Data were digitized at a sampling rate of 1000 Hz using BrainAmp amplifiers (BrainProducts, Munich, Germany), filtered from 0.3 to 120 Hz and downsampled to 250 Hz for the off-line analysis. Epochs were cut around the visual motion onset (0 indicates the first frame of the visible movement) from –1000 ms before to 1200 ms after visual motion onset. Trials containing artifacts in EEG data resulting from eyeblinks, horizontal eye movements, or muscle activity were removed from the further analysis. Noisy channels were linearly interpolated. Finally, an automatic threshold was applied, excluding all trials in which the EEG amplitude exceeded 100 μV.

### Data analysis

Reaction times (RTs) to target stimuli were calculated by averaging all trials in which subjects responded between 230 and 1000 ms after visual motion onset and in which the RT did not exceed 2 standard deviations from the mean RT within each participant and condition. For the statistical analysis of RTs, HR, and false alarms (FA), an ANOVA or Friedman test (if the assumption of gaussianity was violated) with the factor experimental condition (No Interference, Speech Interference, Auditory Noise Interference, Lip Movement Interference) was calculated. A Kolmogorov-Smirnov test was computed to test for gaussianity of RT, HR, and FA distributions. Moreover, three planned contrasts were computed: Speech Interference vs. No Interference, Auditory Noise Interference vs. No Interference, and Lip Movement Interference vs. No Interference.

EEG data were analyzed using MATLAB (Version 7.10), EEGLAB 5.03 (http://www.sccn.ucsd.edu/eeglab), and the FIELDTRIP toolbox (Oostenveld et al., 2011). For the analysis of SSVEPs, event-related activity was calculated by averaging the epochs of each condition. For the averaged activity, TF analyses were calculated using wavelet transformation with Morlet wavelets spanning a range of 10–30 Hz with a length of 12 cycles. The TF analysis was computed in 0.25 Hz steps. In agreement with our previous study (Senkowski et al., 2008), we analyzed SSVEPs for three predefined regions of interest (ROIs): an occipital ROI, comprising of 7 electrodes that were located at midline-occipital scalp, and two symmetric bilateral ROIs that were located at lateral temporal scalp, comprising of 6 electrodes each. In line with the observed SSVEP response pattern, the analysis was done for the time window of 230–550 ms after visual motion onset. To investigate how visual inputs of the center speaker and the flanking speakers were processed in the different experimental conditions, wavelet transformed data were analyzed for those frequencies that corresponded to the visual stimulation frequencies of the speakers. The length of the wavelet was 480 ms for the analysis of 25 Hz activity and 632 ms for the analysis of 19 Hz activity, with a wavelet length of 12 cycles. Repeated measures ANOVAs with the within-subject factors Condition (No Interference, Speech Interference, Auditory Noise Interference, Lip Movement Interference) and ROI (left temporal, right temporal, and occipital) were conducted. Furthermore, planned contrasts between each of the three interference conditions (Speech Interference, Auditory Noise Interference, and Lip Movement Interference) and the no-interference condition were computed. In case of non-sphericity, as tested by Mauchly's sphericity test, the degrees of freedom were adjusted in the ANOVAs.

## Results

### Behavioral data

**Figure 2:**
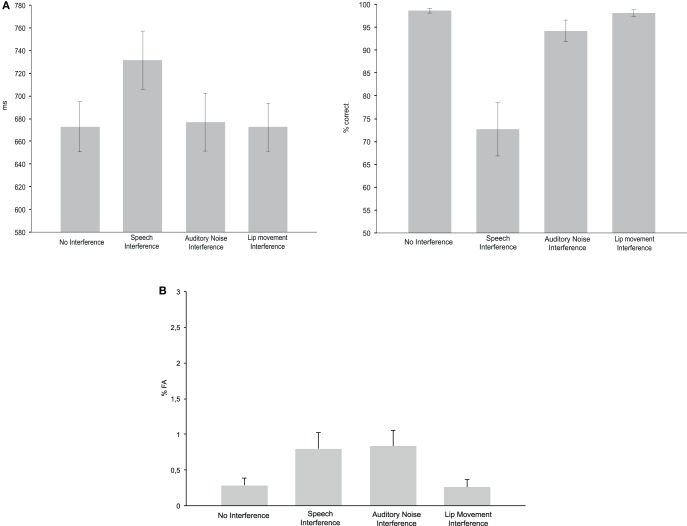
**Behavioral performance. (A)** Reaction times (left panel) and hit rates (right panel) for the No Interference control condition as well as for the three Interference conditions. **(B)** False alarm rates in the four experimental conditions.

The ANOVA for RTs with the factor Condition (No Interference, Speech Interference, Auditory Noise Interference, and Lip Movement Interference) revealed a significant effect [*F*_(2.07, 24.85)_ = 16.169, *p* < 0.0001; Figure 2B]. The analysis of planned contrasts revealed significant longer RTs in Speech Interference Condition (731 ms) compared to the No Interference condition (673 ms; *t*_12_ = −6.557, *p* < 0.001). No other significant effects were observed for RTs.

Since the Kolmogorov–Smirnov tests indicated violations of gaussianity in the distributions of HR and FA data, non-parametric Friedman tests were computed for the analysis of effects in HR and FA rate. For the HR, this test revealed a significant difference between conditions (*p* < 0.0001). The analysis of pair-wise planned contrasts (using non-parametric Wilcoxon tests) revealed significant differences between the No Interference and the Speech Interference Condition (*p* = 0.001) and the No Interference and the Auditory Noise Interference Condition (*p* = 0.003). For both comparisons the HR was higher in the No Interference condition. There was no significant difference between the No Interference and the Lip Movement Interference Condition (*p* = 0.128). For the three Interference Conditions, a significant difference was found between the Lip Movement Interference and the Auditory Noise Interference Condition (*p* = 0.011), due to a higher HR in the Lip Movement Interference Condition. Furthermore, there were significant differences between the Lip Movement Interference and the Speech Interference Conditon (*p* = 0.001) as well as between the Speech Interference and the Auditory Noise Interference Condition (*p* = 0.001). The HR was higher in the Lip Movement and the Auditory Noise Interference conditions compared to the Speech Interference Condition.

The Friedman test for FA rate revealed a significant result (*p* = 0.019; Figure 2B). Pairwise Wilcoxon tests revealed significantly larger FA rates in the Speech Interference Condition (0.799%) compared to the No Interference Condition (0.287%, *p* = 0.021). However, the differences between the No Interference compared to the Auditory Noise Interference Condition (0.835%) and the Lip Movement Interference Condition (0.257%) were not significant.

### Steady-state visual evoked potentials

**Figure 3:**
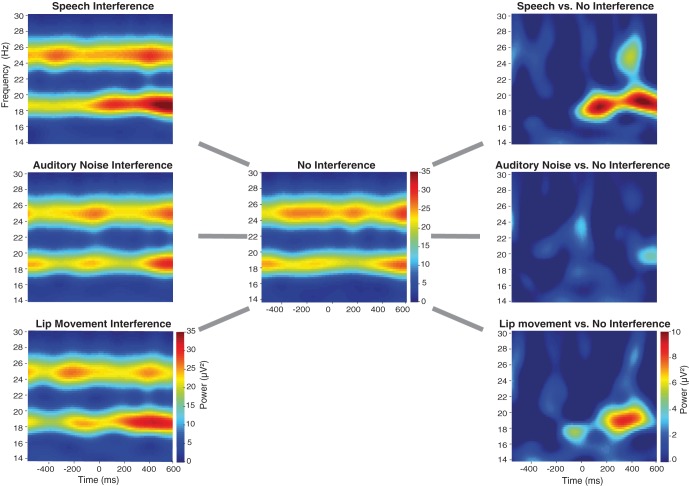
**Time-frequency-plots of SSVEPs for the three Interference conditions (left panel), the No Interference condition (middle panel) as well as for the differences between Interference, and No Interference conditions (right panel) for the occipital ROI (see Figure 4B).** For the statistical analysis a time-frequency window of 230–550 ms and 19 Hz was selected.

**Figure 4:**
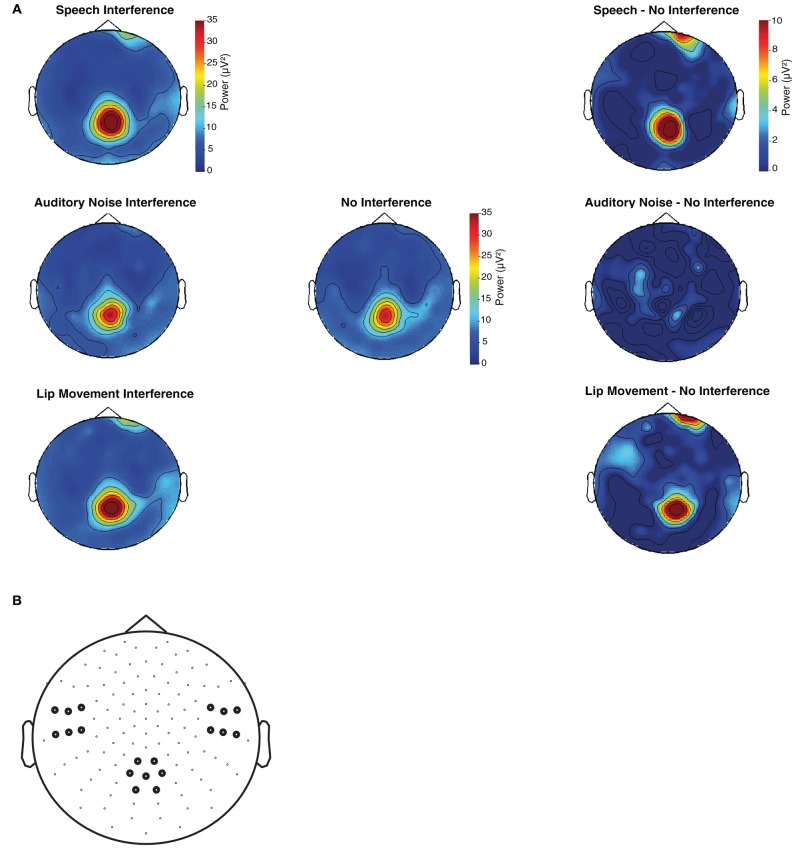
**(A)** Topographies of flanking speaker's induced SSVEPs (19 Hz) for the time window of 230–550 ms after visual motion onset. **(B)** Left temporal, right temporal, and occipital sensors were pooled in three regions of interest and used for the statistical analysis of SSVEPs.

The spectral analysis revealed occipital SSVEPs that corresponded to the flicker frequency of flanking speakers (19 Hz) and the center speaker (25 Hz, Figure 3). The Two-Way ANOVA for flanking speakers' 19 Hz SSVEPs using the factors Condition (No Interference, Speech Interference, Auditory Noise Interference, and Lip Movement Interference) and ROI (left temporal, right temporal, and occipital) revealed significant main effects of Condition [*F*_(3, 12)_ = 4.123, *p* < 0.05] and ROI [*F*_(2, 12)_ = 12.780, *p* < 0.001], and a significant interaction between these factors [*F*_(6, 72)_ = 2.770, *p* < 0.05]. Follow-up analyses were performed separately for the three ROIs. Whereas no significant effects were observed for the bilateral temporal ROIs (all *p*'s > 0.1), a significant main effect of Condition was found for the occipital ROI [*F*_(3, 12)_ = 3.777, *p* < 0.05, Figure 4]. The analysis of planned contrasts revealed a significant effect for the contrast between the Speech Interference and the No Interference condition [*F*_(1, 12)_ = 5.996, *p* < 0.05], due to larger flanking speaker SSVEPs in the Speech Interference condition. Moreover, a trend toward significance was found for the contrast between the Lip Movement Interference and the No Interference condition [*F*_(1, 12)_ = 4.488, *p* < 0.1]. SSVEPs tended to be larger in the Lip Movement than in the No Interference condition. No other significant effects were found, neither in the 19 Hz nor in the 25 Hz SSVEPs.

The present finding of a occipital modulation of the 19 Hz SSVEPs differs from our previous study, which found relevant effects at a left temporal ROI. To ensure that the differences in the topographic distribution of the maximum SSVEP power between our studies are not due to a technical malfunction, we tested the original stimulation setup as used in our previous study (Senkowski et al., 2008) as well as the stimulation files which we used in the present study with a photodiode but found no deviations in visual stimulation frequencies.

## Discussion

The present study demonstrates that processing of distracting audiovisual speech signals interferes with the recognition of attended audiovisual speech. Comparing speech recognition performance in three interference conditions with a no-interference control condition, we observed a decrease in response speed primarily when the distracting signals comprised of naturalistic audiovisual speech. This finding was paralleled by an enhancement of flanking speakers SSVEPs over the occipital lobe.

### Behavioral data

From the three distraction conditions (Figure 1), an interference effect in RT data was found particularly in the naturalistic audiovisual speech interference condition. Although we also found a significant difference in the HR between the Auditory Noise Interference Condition and the No Interference Condition, the most robust interference effects on RT, FA, and HR were observed in the Speech Interference Condition (see Figure 2A). Previous studies have shown that synchronously presented auditory and visual stimuli can serve as salient distractors, which can, for instance, bias temporal order judgements and simultaneity judgements of visual stimuli (Van der Burg et al., 2008a). Furthermore, it has been demonstrated that task irrelevant auditory signals can facilitate visual search (Van der Burg et al., 2011), in particular when the auditory signal is presented synchronously with the visual target (Van der Burg et al., 2008b) and when it is transient (Van der Burg et al., 2010). Using a spatial cueing paradigm, another study showed a stronger attentional capture for bimodal audiovisual compared to unimodal visual distractors (Matusz and Eimer, 2011). All of these studies have used basic, semantically meaningless, auditory, and visual stimuli. A study in which participants were asked to detect or localize a naturalistic face (out of up to four faces) that matches in its lip movements with a simultaneously presented auditory speech, showed a decrease in accuracy and an increase in search times with increasing set size in the localization task (Alsius and Soto-Faraco, 2011). This suggests that the faces were processed in a serial fashion (Wolfe, 2003).

Alsius and Soto-Faraco (2011) conducted another experiment, in which the task was to detect or to localize an auditory stream (out of up to four auditory streams) matching the lip movements of a face. In this experiment, RTs and accuracy did not depend on set size in the detection task, supporting the assumption of parallel processing of the auditory streams. Together, these studies show that auditory speech represents a salient input and that auditory stimuli can strongly bias the processing of concurrently presented visual stimuli.

In the present study two bimodal audiovisual interference conditions were examined: one consisted of natural audiovisual speech signals and the other of lip movements and auditory noise. In agreement with the above-described studies, our finding of distraction effects in the naturalistic speech interference condition suggests that auditory speech stimuli serve as salient inputs in our environment, even if they are unattended. Taken together, our study demonstrates that irrelevant naturalistic audiovisual speech signals have a much stronger interference effect on RTs than visual lip movements alone or lip-movements that are accompanied by acoustic noise. This highlights the unique relevance of speech signals in our environment.

### Interference effects in SSVEP

The finding of enhanced flanking speaker induced SSVEPs for naturalistic audiovisual speech stimuli fits with our previous study (Senkowski et al., 2008), which had only two experimental conditions (Audiovisual Speech Interference and No Interference). Importantly, the present observations extend our previous findings by demonstrating that the enhancement of flanking speaker induced SSVEP occurs primarily when the flanking speakers produced naturalistic audiovisual speech but this enhancement is weaker (in the Lip Movement Interference condition) or even vanished (in the Auditory Noise Interference condition) in the other distraction conditions. In contrast to our previous study (Senkowski et al., 2008), the present results allow a more specific interpretation of the interfering effects of naturalistic audiovisual speech signals, since no interfering effects on RTs were found when auditory noise, which resembled the naturalistic syllables in its basic properties, like stimulus power, was presented. As shown in previous visual attention studies, SSVEP enhancement likely reflects an increased processing of the respective visual flicker stimuli and thus can serve as an electrophysiological measure for the allocation of visual attention (Morgan et al., 1996; Müller et al., 2003; Martens et al., 2011). Therefore, we suggest that the enhanced flanking speaker's SSVEPs reflect a capture of visual attention by the non-relevant audiovisual speech signals.

Another interesting observation was the trend toward a significant enhancement of the flanking speaker's SSVEPs in the Lip Movement Interference condition. Since there were no behavioral interference effects of viewing lip movements alone, the enhanced SSVEPs in this condition do not appear to reflect a behaviorally relevant capture of visual attention. An explanation for the observed trend could be that the lip movements of the flanking speakers were not accompanied by an acoustic stimulus, which may have led to a crossmodal mismatch detection (Arnal et al., 2011) that enhanced visual processing of the flanking speakers.

The absence of interference effects on SSVEPs induced by the center speaker is in line with our previous study (Senkowski et al., 2008). It may be that the capture of attention observed in the Speech Interference Condition involves a split of the attentional focus when the flanking speakers produced bimodal audiovisual syllables. Previous studies have shown that the attentional spotlight can be split (Müller et al., 2003; McMains and Somers, 2004). Specifically, these studies have shown that visual input presented at multiple locations can be monitored in parallel by our attentional system. In the current study, however, such a possible split of the attentional spotlight did not substantially affect the processing of visual input from the attended center speaker.

While the finding that the distraction effects are particularly reflected in flanking speakers SSVEP is in agreement with our previous study (Senkowski et al., 2008), there are also some differences in results. The main difference is that the effects on flanking speakers SSVEPs in our previous study were found at left lateral temporal electrode sites, whereas in the present study we observed modulations at occipital sites. The differences between our previous study and the present work may emerge from differences in experimental setups. The paradigm in the present study consisted of four experimental conditions (including three distraction conditions) compared to two conditions (with only one distraction condition) in our previous study. It is possible that these differences contributed to the differences in results (i.e., topography of effects). Notably, however, the effects in both studies were found particularly for flanking speaker SSVEPs. Interpreting the results in terms of a capture of visual attention, the observation of effects at occipital electrodes in the present study fits well with previous studies showing attention related effects on SSVEPs at postero-occitipal scalp (e.g., Müller et al., 2003).

## Conclusion

Our study demonstrates that non-relevant audiovisual speech stimuli serve as highly salient distractors in the processing of audiovisual speech. The enhanced attentional capture in the naturalistic audiovisual speech interference condition is reflected by a decrease in behavioral performance and an enhancement of flanking speaker induced SSVEPs. The interference effects in the other distraction conditions, comprising of visual lip movements alone and lip movements accompanied by auditory noise, were much weaker or even vanished, respectively. Taken together, our study provides evidence that non-relevant audiovisual speech in particular leads to stronger distraction in speech interference situations as compared to other sensory signals.

### Conflict of interest statement

The authors declare that the research was conducted in the absence of any commercial or financial relationships that could be construed as a potential conflict of interest.
